# Prévalence de la prise d'alcool pendant la grossesse à Lubumbashi, République Démocratique du Congo

**DOI:** 10.11604/pamj.2014.18.135.4680

**Published:** 2014-06-11

**Authors:** Arsène Tshikongo Kabamba, Laurent Kwete Shamashanga, Albert Otshudi Longanga, Zet Kalala Lukumwena

**Affiliations:** 1 Faculté des Sciences Pharmaceutiques, Université de Lubumbashi, République Démocratique du Congo; 2 Université Libre de Bruxelles, Belgique; 3 Faculté de Médecine Vétérinaire, Université de Lubumbashi, République Démocratique du Congo

**Keywords:** Alcool, grossesse, femme enceinte, effets secondaires, alcohol, pregnancy, expectant mother, side effects

## Abstract

En dépit de l'ampleur du risque sur le foetus, l'alcool est sans cesse consommé par les femmes enceintes. L'objectif de ce travail est d’étudier l'usage de l'alcool chez la femme enceinte dans la ville de Lubumbashi, d'examiner les dangers potentiels encourus par le foetus, et enfin de faire des recommandations éventuelles en vue de sécuriser la grossesse et la femme enceinte. Du 22 Août au 11 septembre 2012, 145 femmes enceintes suivies en consultation prénatale à l'Hôpital SENDWE ont été invitées à remplir un formulaire reprenant les informations les concernant, le type d'alcool consommé et la période de consommation. 26,2% des femmes interrogées reconnaissent avoir consommé l'alcool et principalement au deuxième et au troisième trimestre de la grossesse pour diverses raisons. Cette étude montre que des efforts restent encore à déployer à Lubumbashi et particulièrement par le personnel soignant de l'Hôpital SENDWE afin de combattre l'utilisation de l'alcool chez la femme enceinte. Elle met également en exergue l'importance de l'information que le corps médical devrait véhiculer auprès des femmes enceintes sur les effets secondaires liés à la consommation de l'alcool pendant la grossesse.

## Introduction

La consommation d'alcool par les femmes enceintes est un sujet sensible. En cas d'excès occasionnel ou chronique durant la grossesse, l'enfant peut subir de nombreuses agressions susceptibles de provoquer un handicap durable. Certains dommages sont décrits en termes de syndrome d'alcoolisation foetale (SAF), d'effets foetaux alcooliques (EFA) et de « Fetal Alcohol Spectrum Disorder » (FASD) [1–3].

L'alcool est un produit tératogène. Sa consommation même ponctuelle ou modérée pendant la grossesse n'est pas anodine et peut entraîner des risques importants. L'alcool agit sur l'embryon et le foetus notamment sur son système nerveux et son cerveau. Il passe du sang maternel vers le sang du foetus au travers le placenta [4, 5].

Tout au long de la grossesse, il agit directement sur le cerveau du foetus en développement. C'est un toxique extrêmement puissant au niveau du cortex cérébral. Dans ces conditions quel que soit le moment de l'alcoolisation de la femme enceinte, le risque d'atteinte des fonctions cérébrales reste très élevé. En outre une consommation importante d'alcool pendant les trois premiers mois de grossesse peut produire des malformations irréversibles chez le bébé [6].

Notre travail voudrait évaluer la prévalence de la prise de l'alcool pendant la grossesse chez les femmes suivies en consultation prénatale à l'Hôpital Sendwe et la diffusion par le personnel soignant de l'information sur les risques pour le foetus.

## Méthodes

Notre étude est de type transversal. Elle vise à analyser l'utilisation de l'alcool chez les femmes enceintes à différentes périodes de la grossesse. Pour ce faire, un questionnaire a été rédigé et destiné aux femmes enceintes suivies en consultation prénatale à l'Hôpital Sendwe. Celui-ci est un Hôpital de référence situé dans la ville de Lubumbashi en République démocratique du Congo. Il comprend en son sein un département de protection maternelle où le suivi des femmes enceintes est assuré par les infirmières et aussi par un médecin consultant.

Cette étude a été réalisée du 22 août au 11 septembre 2012. Cent quarante-cinq femmes enceintes ont été invitées à répondre à un questionnaire reprenant les informations sur la femme enceinte, le type d'alcool consommé et la période de consommation. Parmi les informations concernant la femme enceinte, nous avons noté le Numéro de la fiche et l'adresse de résidence, l’âge de la femme enceinte, l’âge de la grossesse, l'alcool utilisé ainsi que les différentes raisons pouvant justifier son utilisation. S'agissant de la qualité du corps médical, nous avons cherché à savoir si les femmes enceintes ont reçu les informations des infirmiers et médecin sur les effets secondaires liés à la consommation d'alcool pendant la grossesse.

## Résultats

**Figure 1:**
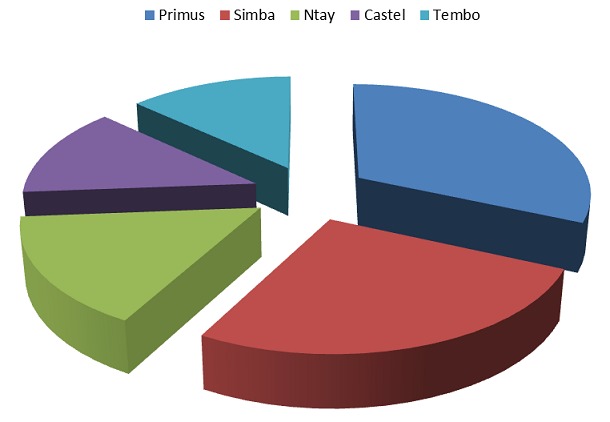
Boissons consommées par les femmes enceintes

Les données sur les femmes enceintes dont les caractéristiques sociodémographiques sont reprises dans le Tableau 1. La distribution selon l’âge des femmes primigestes est mentionnée dans le Tableau 2. La question de la consommation de l'alcool par les femmes enceintes et le type d'alcool consommé, sont repris dans le Tableau 3, le Tableau 4 et la Figure 1.

**Tableau 1:** Caractéristiques sociodémographiques des femmes enceintes interrogées à l'Hôpital SENDWE

Caractéristiques	Effectifs	Pourcentage
**Tranches d’âges**		
16-20	22	15,1
21-30	84	57,9
31-40	39	26,8
**Niveau d’études**		
Primaire	15	10,3
Secondaire	97	66,8
Supérieur	33	22,7
**Occupation professionnelle**		
Ménagères	17	11,7
Elèves	3	2
Etudiantes	5	3,4
Secteur informel	108	74,4
Salariées	12	8,2
**Age de la grossesse**		
**Premier trimestre**		
16-20	9	6,2
21-30	19	13,1
31-40	1	0,7
**Deuxième trimestre**		
16-20	13	9
21-30	32	22
31-40	4	2,8
**Troisième trimestre**		
16-20	11	7,6
21-30	39	26,9
31-40	17	11,7

**Tableau 2:** Distribution des femmes primigestes selon les tranches d’âge

Tranches d'Age	Effectifs	Pourcentage
**Premier trimestre**		
16-20	9	6,2
21-30	16	11
31-40	0	0
**Deuxième trimestre**		
16-20	13	9
21-30	21	14,5
31-40	3	2
**Troisième trimestre**		
16-20	11	7,6
21-30	27	18,6
31-40	0	0

**Tableau 3:** Résultats sur la question de la consommation de l'alcool

Nombre de femmes ayant consommé	38
Raisons	Envie et Appétit
Nombre de femmes ne consommant pas à cause de la connaissance des risques	6
Nombre de femmes ne consommant pas et n'ayant aucune information sur les risques	101
Connaissances sur les répercussions chez les femmes enceintes qui ont consommé	Aucune
Quantité de bouteille en une prise	1bouteille : 30 femmes ; 2bouteilles :6 femmes ; 3bouteilles : 2 femmes
**Age de la grossesse chez les femmes ayant consommées**	
Premier trimestre	0
Deuxième trimestre	23
Troisième trimestre	15

**Tableau 4:** La boisson Alcoolique consommée par les femmes enceintes interrogées à l'Hôpital Sendwe

Type de boisson	Pourcentage de femmes qui ont consommé
Primus^®^	31,6%
Simba^®^	26,3%
Ntay^®^	15,8%
Castel^®^	13,1%
Tembo^®^	13,1%

## Discussion

En nous basant sur les résultats obtenus, nous avons dégagé quelques observations. Cent quarante cinq femmes enceintes ont participé à notre enquête. Elle consistait à étudier l'utilisation de l'alcool pendant la grossesse par les femmes enceintes suivies en CPN à l'Hôpital Jason Sendwe de Lubumbashi. Une étude comparative de données sociodémographiques n'a pas été réalisée dans le cadre de cette enquête, le nombre effectif des femmes enceintes recensées étant très différent dans les différents groupes examinés (tranche d’âge, niveau d’étude, occupation professionnelle etc).

Sur les 145 femmes interrogées, 38 d'entre elles (26,2%) ont reconnu avoir consommé l'alcool soit par envie ou comme apéritif. Le corps médical est constitué en CPN des infirmiers majoritairement. Peu formés, ils ne donnent pas des informations aux femmes enceintes sur les risques accrus de la consommation de l'alcool pendant la grossesse.

C'est dans la tranche d’âge de 21 à 30 ans que nous retrouvons le taux de consommation d'alcool le plus élevé. Ceci est vraisemblablement dû au fait que la plupart de femmes enceintes dans cette tranche d’âge sont des femmes primigestes, peu informées sur les risques d'alcool qu'elles consomment préférentiellement par habitudes. Comme l'indiquent Jacobson et al dans une étude antérieure [4], nous avons aussi observé que les facteurs qui favorisent la forte consommation d'alcool chez la femme enceinte sont nombreux, entre autres une accessibilité facile aux boissons alcoolisées et la dépendance causée par la prise habituelle de ce type de boissons.

C'est au cours du deuxième trimestre et dans une moindre mesure dans le courant du troisième trimestre de la grossesse que nous avons enregistré les taux de consommation d'alcool les plus élevés. Pourtant, c'est pendant toute la durée de la grossesse que l'utilisation d'alcool est formellement contre-indiquée chez la femme enceinte. Les principales raisons de cette consommation sont l'envie et le manque d'appétit. Ces observations rejoignent celles évoquées dans une étude réalisée antérieurement par Nagahara et al [7].

La primus (31,6%) et la simba (26,3%) sont les boissons les plus consommées par les femmes enceintes interrogées (Tableau 3, Tableau 4, Figure 1). L'importante consommation de la primus peut s'expliquer par le fait qu’à la différence des autres boissons alcooliques rencontrées à Lubumbashi, la primus a connu un fort tapage publicitaire. De plus, sa disponibilité et son prix de vente relativement bas la place parmi les boissons alcoolisées les plus facilement accessibles sur le marché congolais. Pourtant, tout alcool, traverse facilement le placenta [8]. Le métabolisme foetal étant immature, il existe un risque de concentrations toxiques. Nous savons par ailleurs que les effets de l'alcool sur le foetus sont nombreux. Une consommation quotidienne d'alcool même très faible ou des ivresses épisodiques pendant la grossesse sont susceptible d'entraîner des complications durant la grossesse (retard de croissance du foetus, accouchement prématuré) ainsi que des troubles psychiques ou du comportement chez l'enfant exposé tels que les troubles d'apprentissages, de la mémorisation, de l'abstraction, de l'attention [9].

Certaines études décrivent comment des troubles du développement des cellules et des organes peuvent ainsi être générés. La perturbation se situe en particulier au niveau de la différenciation de cellules nerveuses entraînant des dommages pour le système nerveux central [1, 10, 11].

Plus l'alcoolémie est élevée, plus les effets secondaires de l'alcool sont importants. C'est le même phénomène pour la femme enceinte et pour son bébé. Comme le placenta ne filtre pas l'alcool, ce produit passe directement du sang de la mère au sang du foetus et le bébé est exposé à la même concentration d'alcool que sa mère.

Par ailleurs, tous les embryons ou foetus exposés à l'alcool ne développent pas nécessairement de problème. Cependant, le risque augmente avec la quantité consommée. La consommation élevée d'alcool entraine le syndrome d'alcoolisation foetale qui a été observé chez des enfants de mères alcooliques. Il est aussi établi que la consommation d'une grande quantité d'alcool chez une femme enceinte augmente les risques d'effets néfastes sur le foetus [5, 10, 12].

En effet, une consommation régulière d'alcool entraine des troubles du développement qui ont aussi été observés chez des bébés nés de mères qui avaient eu une consommation qui aurait été dite à faible risque si elles n'avaient pas été enceintes. La consommation de cinq verres ou plus par semaine augmenterait le risque d'avortement spontanés et de mort nés [13, 14].

Pour ces différentes raisons, nous pensons que les boissons alcoolisées sont à éviter au cours de la grossesse et peut être pris comme une contre indication formelle.

## Conclusion

Les boissons alcoolisées sont utilisées par les femmes enceintes. Les résultats de l'enquête que nous avons réalisée à l'Hôpital SENDWE corroborent cette affirmation. Or, l'utilisation de l'alcool n'est pas sans danger pour l'embryon ou le foetus. Nous pensons que le corps médical devrait accorder une attention particulière à la prise en charge de la femme enceinte. L'information et la formation du corps médical sur les effets de l'utilisation de l'alcool pendant la grossesse devraient être encouragées. L'idéal serait de décourager l'usage de l'alcool chez la femme enceinte au cours des séances d'information destinées aux femmes enceintes pendant les consultations prénatales. Toutefois, les femmes enceintes devraient être suffisamment informées sur les risques encourus et le danger de la prise d'alcool. Un effort particulier devrait être focalisé sur les femmes primigestes dans la tranche d’âge de 21 à 30 ans, car peu expérimentées sur les problèmes gestationnels courants, elles sont sujettes à beaucoup de dérives.
